# Patient-reported treatment burden of chronic immune thrombocytopenia therapies

**DOI:** 10.1186/1471-2326-12-2

**Published:** 2012-03-22

**Authors:** T Michelle Brown, Ruslan V Horblyuk, Kelly M Grotzinger, Axel C Matzdorff, Chris L Pashos

**Affiliations:** 1 RTI Health Solutions, Research Triangle Park, NC, USA; 2 GlaxoSmithKline, Collegeville, PA, USA; 3 Department of Hematology and Oncology, Caritasklinikum Saarbruecken, St. Theresia, Saarbruecken, Germany; 4 United BioSource Corporation, Lexington, MA, USA

**Keywords:** Immune thrombocytopenia, ITP, Burden, Bother, Corticosteroid

## Abstract

**Background:**

Chronic immune thrombocytopenia (ITP) is a debilitating autoimmune disorder that causes a reduction in blood platelets and increased risk of bleeding. ITP is currently managed with various pharmacologic therapies and splenectomy.

This study was conducted to assess patient perceived and reported treatment side effects, as well as the perceived burden or bother, and need to reduce or stop treatment, associated with these side effects among adult patients with chronic ITP.

**Methods:**

A Web-enabled survey was administered to members of a US-based ITP patient support group. Patients reported demographic and clinical characteristics, ITP treatments' side effects for treatments received since diagnosed, level of bother (or distress), and need to reduce or stop treatment, associated with side effects. Current and past exposure was assessed for five specific treatment types: corticosteroids (CS), intravenous immunoglobulin (IVIg), anti-D immunoglobulin (anti-D), rituximab (RT), and splenectomy (SPL), as well as for other patient-referenced therapies (captured as "other").

**Results:**

The survey was completed by 589 patients; 78% female, 89% white, mean age 48 years (SD = 14.71), and 68% reported a typical low platelet count of < 50,000/μL. Current or past treatment with CS was reported by 92% (n = 542) of patients, 56% (n = 322) for IVIg, 36% (n = 209) for anti-D, 36% (n = 213) for RT, and 39% (n = 227) for SPL. A substantial proportion of CS-treated patients reported side effects (98%, *P *< 0.05), were highly bothered by their side effects (53.1%, *P *< 0.05), and reported the need to stop or reduce treatment due to side effects (37.8%, *P *< 0.05). Among patients reporting side effects of treatment, significant associations were noted for the number of side effects, aggregate bother of reported side effects, and the need to stop or reduce treatment (all *P *< 0.05).

**Conclusions:**

Current ITP treatments, particularly corticosteroids, are associated with multiple bothersome side effects that may lead to patients stopping or reducing therapy. Open, informed and complete communication between clinician and patient regarding both the benefits and the side effects of ITP treatment may better prepare patients for their prescribed regimens.

## Background

Immune thrombocytopenia (ITP) is an autoimmune disorder characterized by increased platelet destruction and suboptimal platelet production, resulting in a decreased number of circulating platelets and increased incidence of bleeding [1,2]. The most common symptoms of ITP are mild bruising and mucosal bleeding; however, some ITP patients experience life-threatening epistaxis, menorrhagia, gastrointestinal bleeding, and central nervous system bleeding [3]. In the United States, chronic adult ITP occurs at a prevalence of 9.5-20 per 100,000 persons [4,5]. Although the mortality rate is fairly low in adults (< 1%) under the age of 65 [6]. morbidity increases in older individuals due to age-related spikes in spontaneous bleeding events [7]. and post-splenectomy (SPL) complications [8].

The goal of ITP therapy is to prevent major bleeding. ITP treatment usually consists of corticosteroids (CS) as a first-line approach [9]. Patients intolerant or with contraindications to steroids, are treated with intravenous immunoglobulin (IVIg), and anti-D immunoglobulin (anti-D) [10], either alone or in combination [11]. SPL is frequently recommended as a second-line therapy and results in sustained remission for nearly two-thirds of treated patients [1,12]. Approximately 35-40% of chronic ITP patients are refractory or unresponsive to CS, immunoglobulins, and SPL [1]. For refractory patients, therapeutic options are limited and morbidity increases substantially [7]. Although ITP is not a labeled indication for rituximab (RT), this monoclonal antibody therapy has become an alternative for chronic ITP patients refractory to initial treatments [6]. Complete disease remission has been documented in 25-50% of patients treated with RT, with some patients remaining in remission for more than one year [6,13].

However, safety concerns have also been noted [11,14]. Although standard and emerging therapies have reduced the risk of bleeding among chronic ITP patients, treatments are associated with side effects that may impose substantial burden on patients. The negative effects of long-term CS use have been documented to include diabetes, hypertension, osteoporosis, mood swings, insomnia, weight gain, and increased susceptibility to infection [15]. Clinical trials suggest that IVIg is associated with headache, fever, myalgia, and other immediate effects (as well as rare late effects), while anti-D is associated with chills, pyrexia, increase in bilirubin, and headaches [16,17]. An increased risk of incision site infection and up to one percent chance of post-surgical death from sepsis has been observed among patients undergoing SPL [12,18]. Adverse effects associated with RT infusions include increased susceptibility to infections, progressive multifocal leukoencephalopathy, chills, fever, severe anaphylactoid reactions, and death [6,19].

Although these ITP-treatment-related side effects have been noted, no studies have quantitatively assessed patient-reported ITP treatment side effects along with the real-world burden (i.e., bother or distress) associated with them, or attempted to determine whether their impact is associated with patients reducing or stopping treatment. More generally, few studies have assessed patient perception of ITP therapy and implications for health-related quality of life (HRQL). In one study, Mathias and colleagues [20] developed and validated an instrument to assess the impact of ITP and ITP therapies on patient HRQL. The authors concluded that the symptoms of ITP and side effects of various therapies can have a significant negative impact on patient HRQL [21]. A more thorough understanding of patient perception of treatment side effects can contribute to more effective clinician-patient communications on the advantages and disadvantages of specific regimens, so that patients can be more informed as they embark on their regimen.

Therefore, to understand the nature of patient perceived chronic ITP treatment side effects, the burden that adult patients associate with them, and their possible impact on the reduction or stopping of treatment, we designed and administered a survey to adults with chronic ITP.

## Methods

### Study sample

Study participants were comprised of self-identified adult patients with chronic ITP from among the membership of the Platelet Disorder Support Association (PDSA), a patient support group in the United States. Eligible study patients met the following criteria: diagnosed with chronic ITP, 18 years of age and older, and past or current experience with one or more of the most frequently used ITP treatment types: CS, IVIg, anti-D, RT, or SPL.

### Survey instrument development

Accepted procedures were used to develop and field the survey instrument [22-24]. To draft the initial version, we identified ITP treatment side effects from product literature and package inserts, a review of the published medical literature, and expert clinical opinion. Upon central institutional review board approval, two patient focus groups with four and seven adults respectively with chronic ITP who met the eligibility criteria stated above, were conducted at the 2008 PDSA annual conference. The feedback of these patient focus groups was used to finalize the list of treatment side effects from the patient perspective and to ensure the clarity and accuracy of the instrument. Finally patient cognitive testing was implemented via phone interviews with three adult patients. These patients simultaneously completed the online survey while providing feedback to a trained interviewer on ease of use, clarity, and acceptance of survey content and instructions.

### Survey instrument

The survey instrument was specifically designed to collect patient self-reported data via a secure Internet portal on: (1) the types and numbers of side effects experienced during past and current treatments; (2) the level of burden, alternatively known as "bother" [25] or "distress", that patients' experienced with each reported side effect; and, (3) the patient-perceived impacts or limitations of their disease and its treatments on current daily functioning and treatment continuation. "Bother" is a term common among clinical researchers focused on patient health-related quality of life. Specifically, it refers to the amount of interference or negative impact an effect or condition has on a patient's well-being. "Aggregate bother" refers to overall or average level of the bother across all experienced effects among patients in receipt of the therapy of interest.

The instrument included modules for each treatment class: CS, IVIg, anti-D, RT, and SPL and an 'Other' intervention category. As part of the automatic navigation of the web-enabled survey, patients were allowed to complete only the modules for the treatments with which they had experience. Each treatment module included questions on treatment effects and associated bother for each treatment effect, as well as questions relating to duration of treatment, time since last treatment, and whether or not the patient had to stop or reduce treatment due to one or more of the reported side effects. Three questions related to patient perceived impact of disease on daily life. Several questions addressed clinical characteristics (e.g., typical low and high platelet count in the past 12 months), symptoms (e.g., wet bleeds (nose or mouth bleeds) and dry bleeds (bruising, hematomas, or petechiae) in the past 12 months), and demographic information.

Treatment side effects, if any, were ascertained via a series of closed ended questions, along with an open-ended option to document an "other" effect, which the patient then listed. Patients could specify a treatment effect that was not listed or could respond that no treatment side effects were experienced. For each selected treatment effect, the automated web-survey allowed patients to indicate their level of bother or distress for that effect, using a standard 5-point, fully anchored, Likert scale [25] (1 = "Not bothered at all;" 2 = "Bothered a little bit;" 3 = "Moderately bothered;" 4 = "Bothered quite a bit;" and 5 = "Extremely bothered"). To assess perceived impact of disease on daily life, patients were asked; "How much does your chronic ITP limit you in your: (a) choice of occupation or job, (b) daily activities, and (c) lifestyle." Again, with a standard 5-point, fully anchored Likert scale [26], patients rated their limitation due to disease in one of the following response categories: 1 = "Not limiting at all;" 2 = "Limiting a little bit;" 3 = "Moderately limiting;" 4 = "Limiting quite a bit;" and 5 = "Extremely limiting."

### Data collection

Upon study approval from the Abt Associates Institutional Review Board, a registered central institutional review board, and in accordance with the Declaration of Helsinki, accepted research practice [22,24], and local laws and regulations, the instrument was fielded among patients with chronic ITP. First, patients in the PDSA were presented with an open invitation to participate. If interested, they then provided online consent as part of the web-based survey, before participating in the web-based survey data collection. The survey was conducted via an Internet portal with appropriate security. To control access to the survey and ensure patient confidentiality, each survey invitation included a unique password-protected link. A small honorarium ($20 USD) was provided to each qualifying patient completing the survey. To ensure complete data, the survey was configured to require an appropriate response to each relevant question before proceeding to the next question or step in the survey.

### Statistical methods

Descriptive analyses and independent t-tests comparing means were conducted. Ordinary Least Squares (OLS) and logistic regression analyses were performed to assess the association with aggregate bother, the need to stop or reduce treatment, and limitation on daily life. Models to evaluate the impact of effects on the need to stop or reduce treatment included the number of side effects and aggregate bother as the independent variables and the need to stop or reduce the dose (yes/no) as the dependent variable. Given that each patient had experience with a different combination of treatment types, a term was created to account for all patients in the model assessing limitation to daily life (treatment exposure (0,1) × Aggregate Bother (1-5)). Aggregate Bother was defined as a per patient weighted average of bother scores across their perceived side effects. Perceived impact or limitations imposed by chronic ITP on occupation, daily activities, and lifestyle were each assessed using OLS regression models which included terms for aggregate bother per side effect per treatment (0 = no treatment and 1-5 = bother). For all analyses, two-sided alpha levels of *P *< 0.05 were considered statistically significant. SAS 9.0 was used for analysis.

## Results

The survey was completed by 589 qualifying chronic ITP patients. All surveys were submitted by respondents with no missing data (consistent with the design of the web survey), and all completed surveys were able to be analyzed.

### Patient demographic and clinical characteristics

Table 1 shows patient demographic and clinical characteristics. Of the 589 patients completing surveys, 78% were female, 89% white, and the mean age was 48 years (SD = 14.71). A majority (59%) had been initially diagnosed 5 or more years previously. While 68% of patients reporting a typical low platelet count below 50,000 /μL in the past 12 months, 54% of patients experienced bleeds at a platelet count less than 25,000 /μL. Most (90%) of patients started taking their medication when their platelet counts decreased below 50,000 /μL, and 56% of patients did not exceed a high platelet count greater than 150,000 /μL.

**Table 1:** Patient clinical and demographic information

		All patients
	**n**	**Percentage**

**Total**	589	100%

**Demographics**		

**Age (Mean (SD))**		47.74 (14.71)

18-24	32	5.4%

25-34	96	16.3%

35-44	122	20.7%

45-54	134	22.8%

55-64	133	22.6%

65-74	51	8.7%

75+	21	3.6%

**Race/Ethnicity (multiple response)**		

White, not of Hispanic origin	521	88.5%

Hispanic	27	4.6%

Black, not of Hispanic origin	23	3.9%

Asian or Pacific Islander	12	2.0%

American Indian/Alaskan Native	8	1.4%

**Employment**		

Employed--Full time	284	48.2%

Employed--Part time	77	13.1%

Self-Employed	38	6.5%

Unemployed	190	32.3%

**Patient symptoms and clinical characteristics**		

**Wet Bleeds (Nose or mouth bleeds (in past year))**		

Not at all	317	53.8%

Once or twice	193	32.8%

Once a month or up to 12 times	54	9.2%

More than once a month	25	4.2%

**Dry Bleeds (bruising, hematomas, or petechiae (in past year))**		

Not at all	122	20.7%

Once or twice	149	25.3%

Once a month or up to 12 times	132	22.4%

More than once a month	186	31.6%

Among the patients, 92% (n = 542) had current or past use of CS, 56% (n = 322) IVIg, 36% (n = 209) anti-D, 36% (n = 213) RT, and 39% (n = 227) SPL. Patients reported current or past experience with a mean 2.58 (SD = 1.17) treatment types among the five alternatives. A mean of 3.12 (SD = 1.83) treatments was reported across all treatments listed, including Danazol (17%), cyclophosphamide (8%), vinca alkaloids (7%), azathioprine (6%), and cyclosporine (4%).

### Treatment side effects

**Figure 1:**
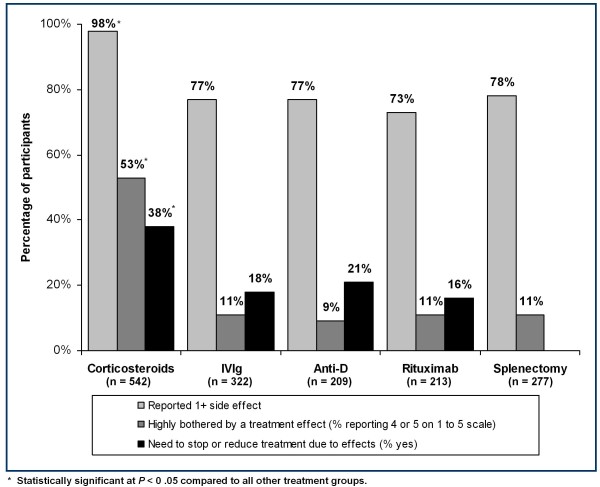
**Reported side effects, highly bothered (% 4-5) and need to stop or reduce treatment by treatment type (% of patients in each category)**.

Patients with current or past CS exposure reported experiencing significantly more treatment side effects with CS exposure than associated with the other four therapies (*P *< 0.05). CS users reported a mean (SD) of 10.7 (5.7) treatment effects, IVIg users had 2.3 (2.2), anti-D patients had 2.4 (2.2), RT patients had 2.1 (2.2), and patients who had received a SPL reported 1.5 (1.2) effects. Fewer patients with CS experience reported no treatment side effects (2%) compared to the other treatment types (*P *< 0.05) ranging from 22% to 27%. As shown in Figure 1, stopping or reducing therapeutic dose (37.8%) was associated with the side effect experience of CS therapy, compared to other types of treatment (IVIg, 18.0%; anti-D, 20.6%; RT, 16.4%. All *P *< 0.05). A high level of burden or bother (4 = "Bothered quite a bit;" or 5 = "Extremely bothered") for one or more side effects of therapy (53.1%) was associated with CS exposure compared to other treatment types (Additional file 1).

### Bother associated with treatment effects

This study reported the number of side effects associated with therapy, the mean bother or distress for each side effect, and a mean aggregate bother score. The percentage of treatment-exposed patients reporting side effects was highest for CS and included: 82.8% of patients reporting weight gain or increased appetite; 77.1%, changes in personality, mood, or emotions, and 75.3%, problems sleeping. SPL also had a highly frequent side effect, scarring from incision (67.4%) with a bother mean of 2.5 (1.22). All other CS identified side effects were reported by fewer than 50% of patients. Among patients who reported one or more treatment effects, mean aggregate bother for CS (3.73 [0.70]; n = 531) was significantly higher than that for IVIg (3.56 [1.00]; n = 247), RT (3.46 [1.03]; n = 153) and SPL (2.74 [1.09]; n = 175) (all *P *< 0.05). The mean aggregate bother for anti-D (3.60 [0.95]; n = 159), based upon patients with one or more effects, was not significantly different than that for CS, although higher than SPL.

### Impact of effects and bother on the need to stop or reduce treatment

Patients who reported one or more side effects were more likely to stop or reduce treatment if they also reported higher aggregate bother for that specific treatment. This was the case for patients undergoing any of the four non-surgical treatment types: CS: OR 1.88 (95% CI 1.40, 2.53), n = 531; IVIg: OR 2.48 (95% CI 1.68, 3.64), n = 247; anti-D: OR 4.21 (95% CI 2.43, 7.31), n = 161; RT: OR 2.88 (95% CI 1.76, 4.72), n = 156. For CS, number of side effects also was significantly associated with the need to stop/reduce treatment (OR 1.05, 95% CI 1.01, 1.08).

### Determinants of treatment type bother

Table 2 shows the results of five regression models that were analyzed to better understand factors associated with aggregate bother among patients with at least one side effect. The models (except for anti-D) were characterized by low adjusted R^2^, suggesting that other factors not in the models also may have contributed to the results. The models did include factors that determined bother, as illustrated by the statistical significance of the beta estimates. Specifically, higher numbers of side effects were significantly associated with increased aggregate bother for all treatment types. The need to stop or reduce treatment also was significantly associated with aggregate bother for CS, IVIg, anti-D, and RT. For those treated with CS, gender also was associated with aggregate bother as female patients reported higher levels of bother than males.

**Table 2:** Regression model predicting aggregate bother among patients with at least one side effect

Independent variables	Corticosteroids		IVIg		Anti-D		Rituximab		Splenectomy	
**Group Mean (SD)§§**	*3.73*	*(0.70)*	*3.56*	*(1.00)*	*3.60*	*(0.95)*	*3.46*	*(1.03)*	*2.74*	*(1.09)*

**Adjusted R^2 ^(N)**	*0.19*	*(440)*	*0.11*	*(208)*	*0.32*	*(128)*	*0.19*	*(130)*	*0.12*	*(151)*

**Age**	0.03(.00)		-0.06(.00)		-0.01(.00)		-0.13(.01)		0.05(.01)	

**Frequency of dry bleeds (0 = No bleeds, 1 = bleeds)**	0.01(.10)		-0.10(.25)		0.24(.26)	**	0.16(.29)		-0.03(.25)	

**Time since diagnosis¶**	-0.01 (.05)		0.00(.12)		-0.01(.13)		0.07(.17)		0.13(.26)	

**Gender (0 = Male; 1 = Female)**	0.17(.08)	****	0.10(.17)		0.09(.20)		0.06(.21)		0.05(.21)	

**Longest cycle duration§**	-0.07(.04)		-		-		-		-	

**Typical low platelet count (past year)**	0.02(.00)		00(0)		0.25(.00)	*	-0.10(.00)		0.14(.00)	

**Need to stop or reduce (0 = No, 1 = Yes)**	0.15(.06)	**	0.31(.16)	****	0.43(.17)	****	0.34(.21)	****	-	

**Number of treatment effects**	0.35(.01)	****	0.15(.04)	*	0.24(.04)	**	0.23(.04)	**	0.34(.09)	****

**Number of ITP treatments (among 5 treatment**	-0.04(.02)		-0.11(.05)		-0.04(.06)		0.09(.07)		-0.01(.07)	

**types)**										

**Other ITP medications in combination (0 = No;**	0.08(.07)		0.07(.15)		0.08(.16)		0.00(.18)		-	

**1 = Yes)**										

**Time since last taking drug/procedure†**	0.08(.07)		-0.02(.15)		-0.18(.19)	*	-0.11(.19)		0.01(.16)	

**Type of physician‡**	-0.06(.09)		-0.03(26)		0.11(.27)		0.07(.40)		-0.02(.24)	

**Frequency of wet bleeds (0 = No bleeds, 1 = bleeds)**	-0.03(.07)		0.07(.15)		-0.22(.16)	**	-0.16(.20)		-0.05(.21)	

* *P <*0.05

* *P <0.01

* **P <0.001

* ***P <0.0001

Each model based on patients reporting 1 or more side effect.

† Categorical variable were 0 = Currently taking (Less than 5 years ago in case of splenectomy), 1 = Taken in the past (5 or more years ago in case of splenectomy)

‡ Type of physician referred to generalist or specialist.

¶ Categorical variable were 1 = 6 months to 1 year ago, 2 = 1 to 5 years ago, 3 = 5 or more years ago.

§ Categorical variable were 1 = Less than 1 month, 2 = 1 to 3 months, 3 = 3 to 12 months, 4 = more than 12 months.

§§ Mean based only on patients who reported 1or more treatment effects for a given treatment type. (i.e., coding of 1 for patients with no side effects was not used).

### Determinants of patients' day-to-day functioning

Greater limitations for occupation, daily activities, and lifestyle were significantly associated with more frequent wet bleeds and lower platelet counts. Greater limitations on daily activities and lifestyle, but not occupation, were significantly associated with more frequent dry bleeds. (Table 3)

**Table 3:** Impact of disease and treatment on patient day-to-day limitation

	Occupation or Job		Daily activities		Lifestyle	
**Group Mean (SD)**	*2.11*	*(1.35)*	*2.25*	*(1.24)*	*2.45*	*(1.25)*

**Adjusted R^2 ^(N)**	*0.17*	*(512)*	*0.17*	*(512)*	*0.18*	*(512)*

**Age**	0.03(.04)		0.12(.04)	**	0.06(.04)	

**AntiD aggregate bother interaction †**	0.20(.05)	****	0.16(.04)	***	0.14(.04)	**

**CS aggregate bother interaction †**	0.12(.04)	**	0.11(.04)	*	0.09(.04)	*

**IVIg aggregate bother interaction †**	0.21(.05)	****	0.15(.05)	**	0.17(.05)	**

**Rituximab aggregate bother interaction †**	0.17(.05)	***	0.12(.05)	*	0.12(.05)	*

**Splenectomy aggregate bother interaction †**	0.20(.05)	***	0.19(.05)	***	0.17(.05)	***

**Frequency of dry bleeds (0 = No bleeds, 1 = bleeds)**	0.05(.05)		0.13(.05)	**	0.15(.05)	**

**Time since last taking drug/procedure§**	0.02(.04)		-0.04(.04)		-0.02(.04)	

**Gender (0 = male; 1 = female)**	-0.06(.04)		-0.06(.04)		-0.05(.04)	

**Typical low platelet count (past year)**	-0.12(.06)	*	-0.12(.06)	*	-0.11(.05)	*

**Number of ITP treatments (among 5 treatment types)**	-0.17(.07)	**	-0.12(.07)		-0.09(.04)	

**Type of physician ‡**	-0.05(.04)		0.04(.04)		0.00(.05)	

**Frequency of wet bleeds (0 = No bleeds, 1 = bleeds)**	0.13(.05)	**	0.13(.05)	**	0.17(.04)	***

* *P <*0.05

* *P <0.01

* **P <0.001

* ***P <0.0001

Each model based on all patients. Patients with no side effects for a given treatment type were coded as 1.

† Interaction term = Aggregate bother (1-5) × treatment received (0,1)

‡ Type of physician referred to generalist or specialist

§ Categorical variable were 0 = Currently taking (Less than 5 years ago in case of splenectomy), 1 = Taken in the past (5 or more years ago in case of splenectomy)

## Discussion

These study findings present new empirical information on patients' perspectives of the burden of ITP therapies: CS, IVIg, anti-D, RT, and SPL. More than two-thirds of the surveyed ITP patients reported experiencing treatment side effects, either at present or in the past. There was a substantial amount of individual patient variation in both the side effects reported by therapy and the magnitude of bother or burden of those side effects.

Although patients who used any of the five ITP therapies were highly bothered or distressed by particular side effects, CS was associated with more side effects and the highest magnitude of bother. Significantly, the proportion of patients treated with CS (37.8%) who had to stop or reduce treatment due to treatment CS side effects was roughly double the proportion of patients treated with other therapy types (IVIg, 18.0%; anti-D, 20.6%; RT, 16.4%. all *P *< 0.05) reporting having to stop or reduce treatment due to treatment side effects. The overall burden of CS for ITP patients is significant, especially given that 90% of surveyed chronic ITP patients received CS as part of their treatment regimen. The substantial burden of CS is largely related to the prevalence of associated side effects and the bother or distress that patients attribute to these side effects. Although patients rated particular side effects as highly bothersome within all treatment types, more than half of patients with CS exposure (53%) reported highly bothersome side effects compared to the other therapies. Approximately, 63.7-82.8% of patients were affected by the five most pervasive and highly bothersome CS side effects. Due to the retrospective nature of this study, data were not collected on the specifics of therapy (e.g., CS dose(s) and duration of therapy). Research should be conducted into the particular effects of specific treatment regimens (e.g., long versus short courses of CS therapy), to determine (as appropriate) how different doses and durations may influence the prevalence and bother of side effects in these patients.

Regression models showed patient aggregate bother for each of the treatment types to be associated with: (1) the number of effects experienced and (2) if these effects contributed to patients having to stop or reduce treatment. This finding suggests that the more side effects patients experience, the more likely they experience "bother", and the more likely they may be to stop or reduce treatment. Length of exposure, i.e. duration of therapy, was not a significant determinant of the bother associated with CS use.

The bother that patients attributed to each treatment type depended upon recollection of past and current treatments. Anti-D was the only treatment type with a significant association between recall (time since last treatment) and aggregate bother. Higher levels of anti-D treatment side effect bother were associated with current anti-D treatment, and lower levels of bother were associated with past treatments. In addition, anti-D was the only treatment type studied in which its patient-perceived bother was associated with additional factors than the number of side effects experienced and the need to stop or reduce treatment. These findings may be partially explained by patients reporting perceived greater bother of side effects than they are presently experiencing. Also specific side effects commonly associated with anti-D (i.e., fatigue, pain, and dizziness) may lend to a greater recall effect--being perceived as more bothersome in the present, than in the past.

A limitation of the study is its focus on five frequently used traditional therapies; patients may have chosen non-traditional therapies to avoid side effects [27]. New therapies, such as the thrombopoeitin (TPO) receptor agonists, were only recently approved in the US at the time of this survey. It may be informative to repeat this research now that the TPO receptor agonists and mimetics are more commonly available in clinical practice. As treatment evolves, it will be important to understand the corresponding patient perspectives [28]. Also, it is not known whether the sample of PDSA members is representative of chronic ITP patients in the community. Although the demographic and clinical characteristics of these patients are similar to those observed in recent publications and consistent with those expected for patients with chronic ITP [3,29], replicating this study in other cohorts would be valuable. Finally, this prospective patient-reported outcomes study depended on patient recall, which may not be as accurate as direct, coincident to therapy, measurement. However, the large sample size and the complementarity of the reported findings provide an indication of the robust nature of the results.

## Conclusions

In conclusion, side effects and associated bother may lead to reductions in treatment adherence and limitations in daily functioning. The burden of CS is especially high, with more than four times the percentage of patients reporting highly bothersome side effects compared to other treatment types, and double the percentage of patients reducing or stopping treatment as a result. Open communication between clinician and patient regarding ITP treatment side effects as well as benefits can help patients become aware of what effects may be experienced on treatment, so that ultimately adherence may be improved.

## Competing interests

TMB and CLP performed this study as employees of Abt Bio-Pharma Solutions, now United BioSource Corporation, which received funding from GlaxoSmithKline to conduct the research. RVH and KMG performed this work as employees of GlaxoSmithKline, which funded this research. ACM serves as an advisor to GlaxoSmithKline, which funded this research.

## Authors' contributions

All authors have made substantive intellectual contributions to this study. TMB contributed to study design, data collection, and analysis, and drafted the initial version of the manuscript. RVH, KMG and ACM also contributed to the study design, analysis, and critical revision of the manuscript for important intellectual content. CLP contributed to study design, data collection, analysis and critical revision of the manuscript. Each author has participated sufficiently in the work to take public responsibility for manuscript content, and has read and approved the final manuscript.

## Pre-publication history

The pre-publication history for this paper can be accessed here:

http://www.biomedcentral.com/1471-2326/12/2/prepub

## Supplementary Material

Additional file 1 **Appendix Table for Editorial Review--Treatment side effects and bother**. Click here for file
