# Supplementary material for: Patient-reported treatment burden of chronic immune thrombocytopenia therapies
Source: BMC Blood Disord. 2012 Mar 22;12:2. doi: 10.1186/1471-2326-12-2 (PMC3350461; doi:10.1186/1471-2326-12-2)
Supplement: Additional file 1 — Appendix Table for Editorial Review--Treatment side effects and bother. [file 1471-2326-12-2-S1.DOC]

## Appendix Table for Editorial Review - Treatment side effects, bother, and weighted bother

|  | Total Treatment | Bother  (among % yes) | | Weighted Bother |
| --- | --- | --- | --- | --- |
|  | % yes | Mean | SD | (%yes X mean) |
| Corticosteroids (total) | n= 542 |  |  |  |
| Weight gain or increased appetite | 82.8% | 4.20 | (1.01) | 3.48 |
| Changes in personality, mood, or emotions | 77.1% | 4.11 | (0.96) | 3.17 |
| Problems sleeping | 75.3% | 4.10 | (0.97) | 3.09 |
| "Moon face" or puffy cheeks | 67.3% | 4.20 | (1.01) | 2.83 |
| Fatigue, tiredness, or weakness | 63.7% | 4.05 | (0.92) | 2.58 |
| Hyperactivity or shakiness | 55.4% | 3.74 | (1.01) | 2.07 |
| Aches and pain in bones, muscles, or joints | 55.4% | 3.94 | (1.16) | 2.18 |
| Swelling or bloating | 54.2% | 3.87 | (1.06) | 2.10 |
| Excessive sweating | 48.0% | 3.76 | (1.03) | 1.80 |
| Hot flashes or flushing | 47.2% | 3.69 | (1.11) | 1.74 |
| Skin problems or changes | 45.9% | 3.59 | (1.14) | 1.65 |
| Confusion or difficulty concentrating | 43.4% | 3.98 | (0.99) | 1.73 |
| Changes in hair and nails | 41.5% | 3.60 | (1.08) | 1.49 |
| Headache | 38.2% | 3.64 | (1.05) | 1.39 |
| Heartburn, stomach, or digestion problems | 37.6% | 3.63 | (1.08) | 1.36 |
| Dry mouth, throat, or voice changes | 31.2% | 3.34 | (1.05) | 1.04 |
| Problems with vision or eye conditions | 30.8% | 3.73 | (0.99) | 1.15 |
| Sexual dysfunction | 30.3% | 3.54 | (1.10) | 1.07 |
| Frequent urination | 25.6% | 3.55 | (1.02) | 0.91 |
| Hump on upper back ("Buffalo hump") | 21.6% | 4.11 | (1.04) | 0.89 |
| Heart or blood pressure problems | 19.4% | 3.91 | (1.00) | 0.76 |
| Infections | 18.5% | 3.72 | (1.06) | 0.69 |
| Blood sugar problems | 17.0% | 4.05 | (1.07) | 0.69 |
| Changes in menstruation | 16.4% | 3.13 | (1.23) | 0.51 |
| Nightmares / night terrors | 15.7% | 3.66 | (1.11) | 0.57 |
| Fever | 11.8% | 3.12 | (1.09) | 0.37 |
| Other | 4.2% | - |  | - |
| None | 2.0% | 1.00 |  | - |
| **Aggregate Bother*** | - | **3.67** | **0.79** | - |
| IVIg (total) | n= 322 |  |  |  |
| Headache | 48.4% | 3.85 | (1.15) | 1.86 |
| Fatigue, tiredness, or weakness | 46.3% | 3.93 | (0.91) | 1.82 |
| Infusion reactions | 32.9% | 3.77 | (1.17) | 1.24 |
| Dizziness or lightheadedness | 27.6% | 3.56 | (1.07) | 0.98 |
| Aches or pain in bones, muscles or joints | 21.1% | 3.94 | (0.94) | 0.83 |
| Infusion site complications | 20.2% | 3.03 | (1.15) | 0.61 |
| Swelling or fluid retention | 15.2% | 3.33 | (1.14) | 0.51 |
| Heart or blood pressure problems | 10.2% | 4.06 | (1.09) | 0.41 |
| Problems with vision or eye conditions | 6.8% | 3.64 | (1.29) | 0.25 |
| Infection | 3.4% | 3.55 | (1.44) | 0.12 |
| Kidney problems | 2.2% | 3.57 | (1.62) | 0.08 |
| Other | 6.2% | - |  | - |
| None | 23.3% | 1.00 |  | - |
| **Aggregate Bother*** | - | **2.97** | **1.39** | - |
| Anti-D (total) | n= 209 |  |  |  |
| Fatigue, tiredness, or weakness | 46.4% | 3.77 | (0.97) | 1.75 |
| Infusion reactions | 41.1% | 4.10 | (1.02) | 1.69 |
| Headache | 37.8% | 3.85 | (1.04) | 1.46 |
| Dizziness or lightheadedness | 27.8% | 3.47 | (1.11) | 0.96 |
| Anemia | 24.9% | 3.73 | (1.14) | 0.93 |
| Aches or pain in bones, muscles or joints | 23.9% | 4.00 | (1.03) | 0.96 |
| Infusion site complications | 12.0% | 2.88 | (1.09) | 0.35 |
| Swelling or fluid retention | 11.5% | 3.71 | (0.91) | 0.43 |
| Heart or blood pressure problems | 7.7% | 3.81 | (1.11) | 0.29 |
| Problems with vision or eye conditions | 6.7% | 3.07 | (1.14) | 0.21 |
| Infection | 2.4% | 3.00 | (1.22) | 0.07 |
| Kidney problems | 0.0% | 0.00 | (0.00) | 0.00 |
| Other | 3.8% | - |  | - |
| None | 23.0% | 1.00 |  | - |
| **Aggregate Bother*** | - | **2.98** | **1.39** | - |
| Rituximab (total) | n= 213 |  |  |  |
| Fatigue, tiredness, or weakness | 47.4% | 3.61 | (1.07) | 1.71 |
| Infusion reactions | 33.3% | 3.87 | (1.12) | 1.29 |
| Headache | 31.5% | 3.52 | (1.12) | 1.11 |
| Dizziness or lightheadedness | 21.1% | 3.60 | (0.94) | 0.76 |
| Aches or pain in bones, muscles or joints | 16.4% | 4.20 | (0.87) | 0.69 |
| Infusion site complications | 13.6% | 3.03 | (1.15) | 0.41 |
| Swelling or fluid retention | 11.7% | 3.68 | (0.95) | 0.43 |
| Anemia | 10.8% | 3.70 | (1.22) | 0.40 |
| Heart or blood pressure problems | 8.0% | 3.94 | (0.97) | 0.32 |
| Problems with vision or eye conditions | 7.0% | 3.60 | (1.30) | 0.25 |
| Infection | 4.7% | 3.60 | (1.17) | 0.17 |
| Kidney problems | 1.4% | 4.00 | (1.00) | 0.06 |
| Other | 4.7% | - |  | - |
| None | 26.8% | 1.00 |  | - |
| **Aggregate Bother*** | - | **2.77** | **1.41** | - |
| Splenectomy (total) | n= 227 |  |  |  |
| Scarring from incision | 67.4% | 2.50 | (1.22) | 1.69 |
| Numbness at the site of incision | 40.5% | 2.58 | (1.04) | 1.04 |
| Prolonged or repeated use of antibiotics | 16.3% | 3.73 | (0.99) | 0.61 |
| Infection | 13.7% | 3.87 | (1.12) | 0.53 |
| Surgery complications | 13.2% | 4.30 | (0.99) | 0.57 |
| Other | 8.4% | - |  | - |
| None | 21.6% | 1.00 |  | - |
| **Aggregate Bother*** | - | **2.34** | **1.21** | - |

* Mean based on all patients exposed to treatment
